# Pediatrics

**Published:** 1906-10

**Authors:** Maud J. Frye

**Affiliations:** Buffalo, N. Y.


### PROGRESS IN MEDICAL SCIENCE.

### Pediatrics

Conducted by MAUD J. FRYE, M.D., Buffalo, N. Y.

### LEUCOCYTOSIS IN WHOOPING COUGH.

CHURCHILL (*Jour. A. M. A.*, May 19, 1906) reviews the literature on this subject and adds the results of his own investigations, 36 cases having been studied. The patients ranged from 6 mos. to 17 yrs. The total leucocyte count was made in 29 cases. A general leucocytosis was found in all but one. The counts range from 10,000 to 112,000. Differential counts were made in all cases, 30 showing a lymphocytosis; 15 out of 16 patients examined in the catarrhal stage had a lymphocytosis. The percentages ranged from 34.3, in a 5 year old child, to 93 in a 4 year old. The latter was the one with a total of 112,000, was a severe case, and came to the clinic at the height of the disease. A count made 16 days later showed a total of 32,600 with 64 per cent. of lymphocytes, and great improvement in all the symptoms.

The total number of cases studied upon which the author bases his deductions was 100. The following conclusions summarize the paper:

1. A general leucocytosis is present in almost all cases of whooping-cough.

2. A lymphocytosis, i. e., an increase in the number of lymphocytes is found in about 85 per cent. of cases at some time during the course of the disease.

3. A lymphocytosis is found even more constantly during the early or catarrhal stage, over 90 per cent. showing the phenomenon at this time.

4. A lymphocytosis is found usually in those conditions difficult to distinguish from whooping-cough.

5. Therefore, the presence of a lymphocytosis in a child with a hard persistent cough is a factor of great diagnostic value. It is also of prophylactic importance inasmuch as it can be utilised to prevent the spread of the disease by leading to the prompt isolation of the patient.

6. The child's age must be taken into account in estimating the importance of the lymphocyte percentage.

MELENA NEONATORUM.

Blount and Gardner (*Am. Jour. of Obstetrics*, Feb., 1906) report a case of melena neonatorum developing four days after birth in a male child weighing 10¼ lbs. Hemorrhage began from the roof of the mouth followed by bloody stools. The hemorrhages from the bowels were repeated every two to six hours for 56 hours. It was estimated that one pint of blood was lost in all.

The treatment first consisted of gelatine solution by mouth, an ounce of a two per cent. solution every two hours. At the same time an ounce was given every four hours by rectum. Later the gelatine by mouth was discontinued on account of its being promptly followed by griping and bloody stools. The gelatine per rectum was continued for 36 hours. Adrenalin solution (1-1,000) M. I. every hour was also given. Morphine was given to control restlessness. On the morning of the third day the breath, bowel movements and gases were very foul, the temperature was 101.6°, the child's condition grave, the stools containing more blood than at any previous time. All former treatment was suspended and the bowel was irrigated every 4 hours with lime-water and salt solution in equal parts, half an ounce of fluid being allowed to remain in the bowel. One ounce of lime-water was given by mouth three times daily. There were no further hemorrhages after this time, the temperature fell to subnormal and recovery was rapid. The birth weight was regained in four weeks.

### GLANDULAR FEVER.

VIPOND (*Archives of Pediatrics*, January, 1906) writes of this disease and reports twelve cases from his own practice. He describes the disease as follows:

Glandular fever is a disease which presents distinct signs and symptoms, and, as a rule, is not difficult to diagnose when developed. It is a disease of childhood and is rarely seen after sixteen. Children between four and twelve years are most frequently attacked. It occurs in narrowly limited epidemics, but if one child in a family contracts the disease the rest are liable to be affected. Neuman claims that the disease is most likely due to streptococcus infection, the streptococcus entering by way of the tonsil without producing any local lesion. Dr. Koplik suggests that infection may be by way of the thoracic duct. It attacks both sexes in equal proportions. Most cases are found during the damp, cold months.

The incubation is from five to seven days. The onset is sudden, the child complaining of headache, pains in the limbs, vomiting, pain in abdomen, chills and loss of appetite. The bowels are constipated, but rarely there is diarrhoea. The tongue is coated, the face flushed. The evening temperature is 102° to 104° F., while the morning temperature is about one degree and a half lower; pulse 110 to 130. So far there is nothing characteristic about the symptoms—they may point to influenza, or to one of the acute exanthemata. In twenty-four to forty-eight hours' time the patient complains of stiffness and pain in the neck, the pain being aggravated by movement. On making an examination of the neck we find that the lymph nodes are enlarged, but on inspecting the throat we find it to be quite free from any signs of diphtheria or other inflammatory condition, and at the most some redness of the tonsils is found. In only one of my 12 cases did I detect any sign of pharyngitis; the condition of the lymph nodes is quite characteristic, and when it develops any doubt about the diagnosis is set at rest. Usually the lymph nodes on the left side of the neck are first involved, the nodes at the posterior border of the sternomastoid being first affected. They are hard, intensely tender when handled, and freely movable, about the size of a large bean, and form a regular chain down the neck. Soon the lymph nodes at the angle of the jaw, and at the anterior border of the sternomastoid become large and tender, and may reach the size of a large marble; when three or four of the nodes in this region are affected, the swelling is considerable. In one or two days' time the right side of the neck becomes implicated, the order of march the reverse of that on the left side. That is, the swelling begins at the angle of the jaw, and at the anterior border of the sternomastoid; later on the lymph nodes at the posterior border of that muscle become inflamed. The patient is fairly comfortable while at rest, but the slightest movement of the head causes severe pain. The parotid glands are not affected, there is no pain on swallowing, but a sensation of tightness is felt in the throat. The skin covering the lymph nodes is in a normal condition.

This disease appears to be a general lymph glandular infection, and rarely do we find the nodes in the neck alone involved. The axillary and inguinal lymph nodes appear to be implicated in most cases. All of my cases presented large nodes in the axillary or inguinal regions, but more frequently in both. The axillary and inguinal lymph nodes were affected in 75 per cent. of Park West's cases. The question arises, Are the mesenteric and the tracheo-bronchial lymph nodes involved? Comby states that it is not demonstrated. Park West claims that the mesenteric nodes were enlarged in 37 of his cases. Several of my patients suffered severe abdominal pain and tenderness on examination, and I have not the least doubt but that the mesenteric lymph nodes are involved in a large number of cases. In not one of my 12 cases did I detect any signs such as paroxysmal cough or difficulty in breathing pointing to the implication of the tracheo-bronchial lymph nodes. Park West found the spleen to be enlarged in 57 of his cases. Seibert reports no enlargement of the spleen, and I might add that I noticed it in only one of my cases. The liver was enlarged in 90 per cent. of the cases reported by Dawson Williams, but there was no enlargement of the liver in any of my patients.

The evening temperature remains high for three to seven days with morning remissions, and frequently ends by crisis with a profuse perspiration. The first sign of improvement noticed is in the fall of temperature, and at the same time the lymph nodes are not so tender, and the neck can be moved without causing great pain, the swelling begins to subside, but this is a slow process, and we cannot generally expect complete resolution until fourteen to twenty-one days, and even after three weeks' time some swelling may still be detected. As soon as the temperature falls the little patient feels and looks better, the appetite returns and convalescence is soon complete. Do the lymph nodes always resolve or does suppuration ever take place? Neumann detected suppuration in 13 of his cases. Comby states that it is a rare event, and I am inclined to think that it does not take place in typical glandular fever. When suppuration is found we must look for some other local source of infection.

The above description refers to an ordinary sharp attack. Many cases present less acute symptoms. The onset is not so abrupt, the evening temperature being about 101° ; and the disease lasting only two or three days. Resolution is just as prolonged.

The prognosis is good. The complications are not numerous. Epistaxis has been noticed, also nephritis and hematuria.

The treatment is supportive. Locally the author has used with good results iced bichloride compresses. During convalescence tonics are indicated.

### ACUTE AFFECTIONS OF THE PHARYNGEAL TONSIL.

DUPUY (*New Orleans Med. and Surg. Journal,* March, 1906) says: While the subject of hypertrophy of the pharyngeal tonsil has been well-nigh exhausted by voluminous contributions, one phase of the question has been completely ignored by the older observers, and is scarcely given its deserved attention by the present-day clinician. We are all familiar with the symptom complex of chronic enlargement of the tonsil, for many of us have come to regard it as a pathological condition originating rather abruptly instead of one resulting from acute inflammatory attacks. It is precisely the acute inflammations of the adenoid which constitute an interesting chapter in the diseases of children.

For practical and clinical guidance the pathology of the pharyngeal tonsil is exactly that of the faucial. The morbid alterations which occur in acute inflammation of the latter, and which can be rightly interpreted even by the medical tyro, are repeated in every particular when the adenoid reacts to microbic invasion. The tendency of infant lymphoid tissue to take on inflammation explains why this tonsil is so frequently attacked. After each attack there results a gradual increase of the glandular and connective tissue elements which leads ultimately to a permanent enlargement. A vicious circle is thus established, as a chronic enlargement of the pharyngeal tonsil again invites recurrent attacks of inflammation. It is now accepted that both in the faucial cavity and in the naso-pharynx the tonsilar inflammation is of infectious origin, the staphylococcus pyogenes being the organism chiefly concerned in the production of these affections. On the other hand, such dietetic influences as arise from the existence of lymphatism and uric acid, etc., must weaken local resistance, acting therefore as contributing factors. Gastro-intestinal disturbances are known to produce congestion of the whole upper respiratory tract, including the adenoid, which, under such irritation, becomes turgescent and is thus prepared to take on active pathological changes.

The clinical picture is that of a head cold with mouth breathing, free nasal discharge, some fever, extreme restlessness. Examination discloses open and normal nasal cavities. Faucial tonsils not visible. Pharynx healthy-looking; when tongue is depressed, which causes gagging, a large quantity of muco-purulent secretion is squeezed from the naso-pharynx and passes down the pharyngeal wall. Lungs normal, no cough and not the slightest evidence of gastro intestinal trouble. This is one of the common minor ailments of childhood and is wrongly considered a head cold, if by that is meant a rhinitis pure and simple. Primary nasal affections are extremely infrequent at this period.

The clinical picture which I have outlined above offers variations according as the middle ear or the lower respiratory tract becomes involved. The eustachian tube being shorter and wider in the earliest years is an anatomical feature which in itself favors the passage of pathogenic organisms from the post-nasal space into the middle ear. It is therefore quite evident that an inflamed adenoid is a real menace to the ear. Deafness and otalgia occur quite frequently in such a condition. On the subsidence of the acute affection these phenomena disappear. The character of the adenoid inflammation, the virulency of the infection, will determine whether or not the disturbance culminates in an acute suppuration. By proper treatment, therefore, of the tonsilar affection we can oftentimes avert the extension of infection to the tympanic cavity and then diminish the possibilities of chronic suppurations which are now rightly considered to be the outcome of acute otitis.

In many instances the secondary phenomena appear in the lower respiratory tract, where the effect is manifested either as spasmodic croup or a simple laryngitis, or as an acute bronchitis.

A smaller number of cases do not present symptoms pointing to the naso-pharynx. A septic temperature and general depression, a languid irritable child, and no lung or gastro-intestinal symptoms.

The treatment consists essentially in post-nasal irrigations with alkaline antiseptic solutions. At this early period we are compelled for evident reasons to reach the naso-pharynx by way of the nasal chambers—either a syringe or a good spray being used. I have a preference for the spray. With the child's head inclined forward, the danger of forcing septic material into the middle ear in my experience is very remote. Ichthyol ointment—say from 20 to 30 grs. to the ounce—is prescribed for home use. Its application in the nostrils guarantees that it will find its way to the inflamed adenoid.

Argyrol, 10 to 15%, in aqueous solution can be dropped into the anterior nares as the child's head is tilted backward. Its antiseptic and yet non-irritant properties have rendered me good services in post-nasal disinfection.

Appropriate systemic treatment is particularly addressed to any diathetic condition which might exist. Iodide of potash is undoubtedly effective in reducing acute lymphatic enlargements. Remember that the adenoid is a lymphatic. Chloride of potash, internally, watching its action on the kidneys, has its advocates, who declare it a specific. Several experiences have led me to believe that by treating the acute affection of the adenoid we prevent chronic enlargement. This, in itself, is no mean victory for a chronic hypertrophy, passes at once into the field of surgery.

